# Mucosal Melanoma of the Hard Palate: Surgical Treatment and Reconstruction

**DOI:** 10.3390/ijerph18073341

**Published:** 2021-03-24

**Authors:** Stefano Bondi, Alessandro Vinciguerra, Alessandra Lissoni, Nathalie Rizzo, Diego Barbieri, Pietro Indelicato, Silvio Abati

**Affiliations:** 1 Otorhinolaryngology—Head & Neck Surgery Department, San Raffaele Hospital, University Vita-Salute, 20100 Milano, Italy; vinciguerra.alessandro@hsr.it (A.V.); barbieri.diego@hsr.it (D.B.); indelicato.pietro@hsr.it (P.I.); 2 School of Medicine, Vita-Salute San Raffaele University, 20100 Milano, Italy; lissoni.alessandra@hsr.it (A.L.); abati.silvio@hsr.it (S.A.); 3 Department of Dentistry and Stomatology IRCCS San Raffaele Hospital, University Vita-Salute, 20100 Milano, Italy; 4 Pathology Unit, Division of Experimental Oncology, IRCCS San Raffaele Scientific Institute, 20100 Milano, Italy; rizzo.nathalie@hsr.it

**Keywords:** submental flap, oral lesions, mucosal melanoma, oral cavity, follow-up

## Abstract

Mucosal melanomas of the head and neck region are uncommon pathologies that can affect the oral cavity, and are characterized by a high rate of mortality. Considering the lack of knowledge regarding risk and prognostic factors, current best clinical practice is represented by a large surgical excision with disease-free margins, eventually associated with a reconstructive flap. Indeed, given the frequent necessity of postoperative radiotherapy and fast healing process, a reconstruction of the surgical gap is advisable. Even if several flaps have been most commonly used, the submental island flap represents a valid alternative thanks to local advantages and similar oncologic outcomes compared to free flaps.

## 1. Introduction

Mucosal melanoma (MM) represents 1.3% of all melanomas with an aggressiveness that is inevitably associated with poor prognosis, given a five-year survival rate of 23% in patients aged 25–64 years. The evidence of early distant metastasis and the high rates of treatment failure are the reasons for dismal outcomes. Primary mucosal melanoma of the oral cavity (POMM) is considered as a head and neck tumor in National comprehensive Cancer Network (NCCN) guidelines and as a very rare disease with an incidence of 0.2 per million/year [1]. The rarity of POMM is clearly underlined in a recent review on oral MM, which included less than 200 citations and where most information was from single cases or small case series [2]. In recent years, many developments have been made in head and neck surgery and radiotherapy, although no increase in survival has been noted, primarily due to the poorly understood pathogenesis of MM and lack of identification of specific risk factors [1,2,3,4,5,6,7]. Melanocytes are pigment cells with the main role of ultraviolet protection and skin pigmentation. However, these cells are also present in many sun-shielded sites, such as the oral cavity, where their function is not clearly understood, but there is evidence supporting an antimicrobial and immunological activity [8,9]. Proliferation of atypical melanocytes at the interface between the epithelium and connective tissue is presumed to be the origin of MM; moreover, exposure to inhaled carcinogens such as tobacco could provoke the malignant transformation of those atypical melanocytes [1,10,11]. In fact, while some molecular alterations in genes such as *c-KIT*, *BRAF*, and *NRAS* have been found, their presence is extremely variable; nevertheless, cigarette smoking has been considered a risk factor because it usually promotes pigmented oral lesions and their potential transformation into malignant ones [11].

As far as clinical signs and symptoms are concerned, POMM is usually diagnosed relatively early compared with other head and neck MMs thanks to the great accessibility of the oral cavity for inspection [12,13,14,15,16]. In particular, oral MMs currently involve the mucosa of the hard palate and maxillary or mandibular gum [17], while they are extremely rare on the tongue or floor of the mouth. When present, the involved oral mucosa presents hyperpigmented lesions of different colors that can range from brown-black to reddish-white and may present nodular or macular morphology [18]. A macular lesion is flat with a radial growth that foreruns vertical growth and is typical of a long history of melanosis [19]; a nodular lesion is irregular, exophytic, sometimes ulcerated, and associated with worse prognosis due to vertical growth. MMs are classified into five different types, according to the presence of pigmentation and pattern of growth (nodular or macular) [20]. This classification is useful to predict oncologic outcomes: even if the risk of nodal involvement is 25–43% [21] or even higher if the lesion is larger than 4 cm or with a depth of infiltration greater than 5 mm [22], a nodular pattern is associated with higher risk of nodal involvement and consequent worse prognosis [23].

When diagnosed, MMs need proper radiologic work-up to better define the dimension and potential infiltration of adjacent structures [1]. MRI represents the imaging modality of choice in suspicion of MM and its signal depends on the amount of melanotic pigment within the lesion, which gives a typical MRI pattern: T1 hyperintensity and T2 hypointensity [24]. Due to the aggressiveness of the disease, bone erosion, perineural spread, and depth of submucosal infiltration should be analyzed carefully in the pretreatment setting, which, if necessary, can be achieved with maxilla–facial computed tomography (CT). In addition to this, a proper regional and systemic staging of the tumor is usually required and achieved with total-body CT; distant metastases at diagnosis are not common (less than 5–10% of cases), with no differences between oral and other MMs [25] but with typical localizations in the brain and lungs [1].

To reach a precise diagnosis, an incisional biopsy is usually necessary for histological examination and is based on immune histochemical biomarkers such as S-100, HMB-45, melan A, and vimentin; moreover, histologic analysis can add useful information, such as lymph vessel invasion and blood vessel invasion, which are associated with worse prognoses. All the above-mentioned information leads to the staging of the pathology based on the TNM system: however, due to the aggressiveness of the disease, involvement of epithelium/submucosa without nodal metastases is directly classified as stage III; deep infiltration of bone, nerves, skin, or nodal involvement is considered stage IV. As a consequence, stage I and II is not possible in the event of an MM.

Once a diagnosis is made, surgery is considered the primary treatment for POMM, since radical tumor resection with disease-free surgical margins has an essential role in defining the prognosis [26]. Elective neck dissection is advised for lesions arising in the oral cavity [27,28], although some authors have suggested elective neck dissection only in cases of nodular MM or macular MM larger than 4 cm [23], or when the thickness of these lesions is >5 mm since this increases the likelihood of lymph node metastases [29].

In addition to surgery, two options are generally considered: radiotherapy, which is usually applied since it has been shown to increase loco-regional control, although with no increase in long-term survival; chemotherapy is not standardized due to the conflicting data available [3]. Furthermore, very few cases of MM have been treated with carbon ion radiotherapy, which is effective against radioresistant tumor, and preliminary results seem promising [29].

This article aims to report a case of MM of the oral cavity and to review the available literature on this topic, focusing on surgical treatment and reconstruction.

## 2. Case Report

**Figure 1:**
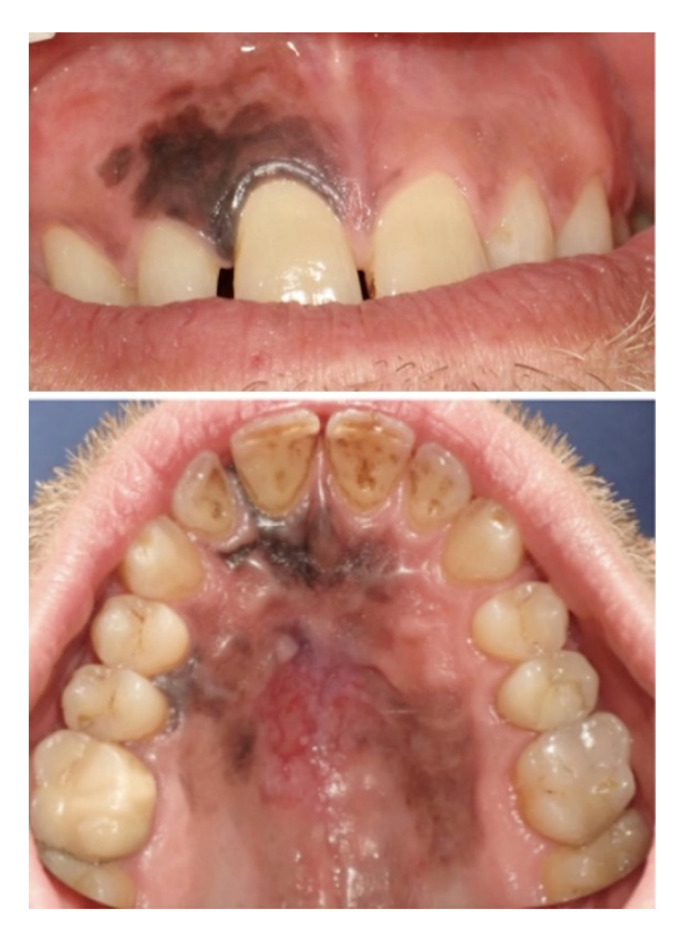
Clinical pictures of the oral pigmented lesions at the first oral pathology visit. Upper gingiva and palatal view.

**Figure 2:**
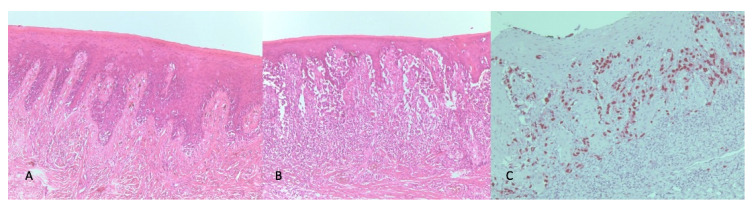
Histopathological images of the biopsied melanocytic lesion of the palate. (**A**) Hematoxylin–eosin staining 50× with evidence of lentiginous junctional melanocyte; (**B**) Hematoxylin–eosin staining 100× with evidence of atypical epithelioid cells; (**C**) Neoplastic cells highlighted at 100× with SOX-10.

A 45-year-old Caucasian man was referred to the Head and Neck Department at San Raffaele Hospital in 2018 with pigmented lesions of the maxillary gum that expanded up to the hard palate. He reported that a small pigmented macule had appeared on the maxillary gum, near the right central incisor, five years before. At that time, his dentist performed a biopsy that reported a benign melanotic macula. In April 2018, a new ulcerated pigmented lesion appeared on the central part of the hard palate, and upon examination in the Oral Pathology Unit, the maxillary gum macula was enlarged, involving the mucosa of both sides of the upper gum and hard palate. In particular, the anterior labial gum pigmentation extended interdentally and became continuous with a large black pigmented lesion in the hard palate, with a central ulceration (Figure 1). A new biopsy was then taken, with a histologic section showing a mucosal lentiginous malignant melanocytic lesion (*S100+*, *SOX 10*+), infiltrating and focally submucosal (Figure 2).

**Figure 3:**
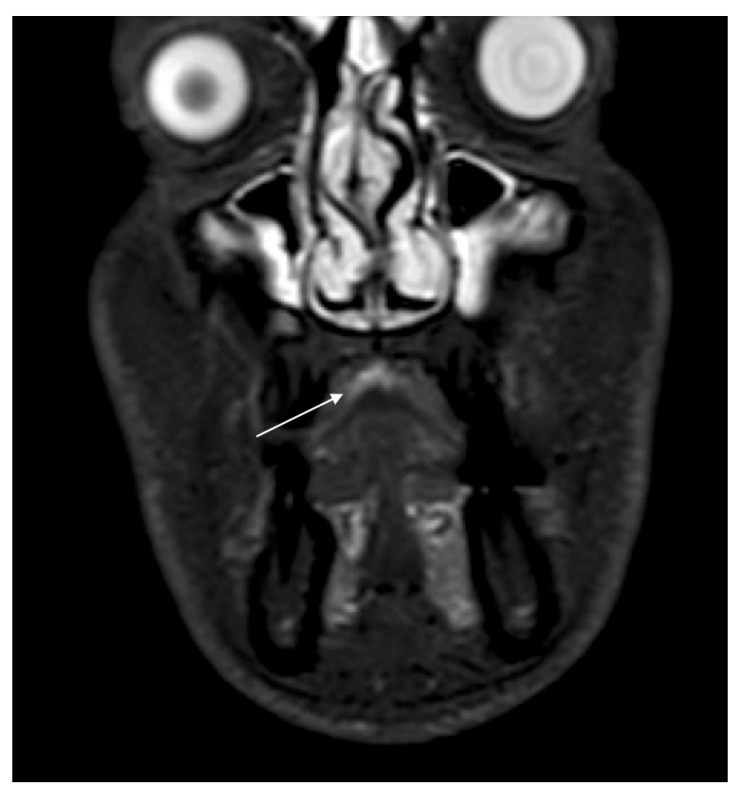
Maxilla–facial MRI in T1WI revealed, in the central area of the hard palate, a thickening of the mucosa of 3–4 mm, with no apparent bone infiltration.

**Figure 4:**
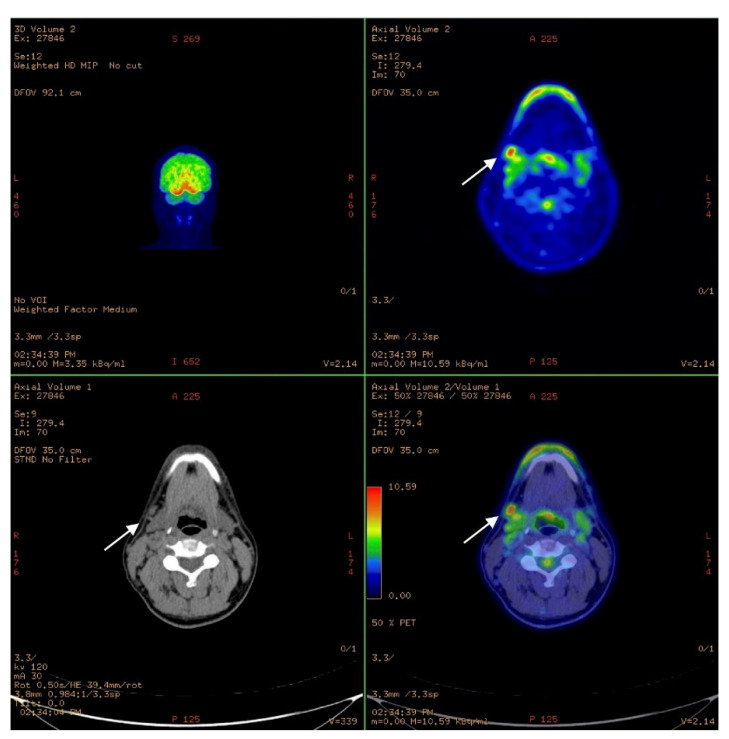
PET/CT (Positron emission tomography/Computed tomography) scan shows positive cervical lymph nodes at level IIa (white arrows).

**Figure 5:**
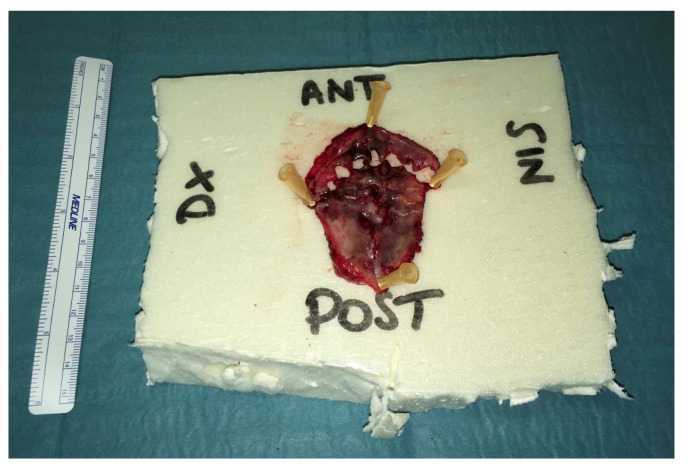
Resected mucosa of the hard palate and maxillary gum with macroscopic evidence of pathological tissue in the alveolar ridge proximal to the melanotic lesion; in addition, seven teeth adjacent to the lesion were removed in order to permit complete resection of pathological tissue. Ant = anterior, Post = posterior; Dx = right; Sin = left.

MRI showed an increased signal in both T1WI and in T1 C+ in the central area of the hard palate with thickening of the mucosa by 3–4 mm (Figure 3). No bone infiltration was seen; on the other hand, PET/CT (Positron emission tomography/Computed tomography) revealed positive cervical lymph nodes, but no distant metastasis (Figure 4). TNM staging was cT3, cN1, cM0 stage IV. Histological mapping before major surgery was performed, and samples, taken from the mucosa of the hard palate and maxillary gum, were positive for MM in situ, with two areas of infiltrating MM in correspondence with the central part of the hard palate and upper gum. The case was discussed with the multidisciplinary team (MDT), which agreed with a program of transoral hard palate–upper gum mucosectomy associated with resection of a cuff of the bone of the upper dental arch (from 1.5 to 2.3 teeth) and the central hard palate, where the invasive MM was previously mapped (Figure 5). Bilateral MRND (modify radical neck dissection) and temporary tracheotomy were also performed. Theoretically, no reconstruction would have been needed because second-intention healing of oral mucosa was possible. Nonetheless, the MDT recommended postoperative radiotherapy (PORT) that cannot be done on demucosized bone, and which is done within 6 weeks after surgery. The reconstruction of surgical gap was performed successfully with a hybrid reverse-flow submental island flap (SIF). In particular, the upper gum–hard palate osteo-mucosal defect was restored with a hybrid reverse-flow SIF with facial nerve-sparing: the facial artery was cut in the proximity of the mandibular branch of the facial nerve, the flap was tunnelized under the nerve and through the buccinator muscle up to reach the cheek mucosa just in front of the Stensen duct papillae. Venous drainage was restored through the interpositioning of the venous graft of external jugular vein between facial vein abutments with a double microanastomosis.

Microscopic examination of the surgical sample confirmed the diagnosis of infiltrating MM of the hard palate; bone and lymph nodes were free of disease. Pathological TNM staging was pT3, pN0 stage III.

**Figure 6:**
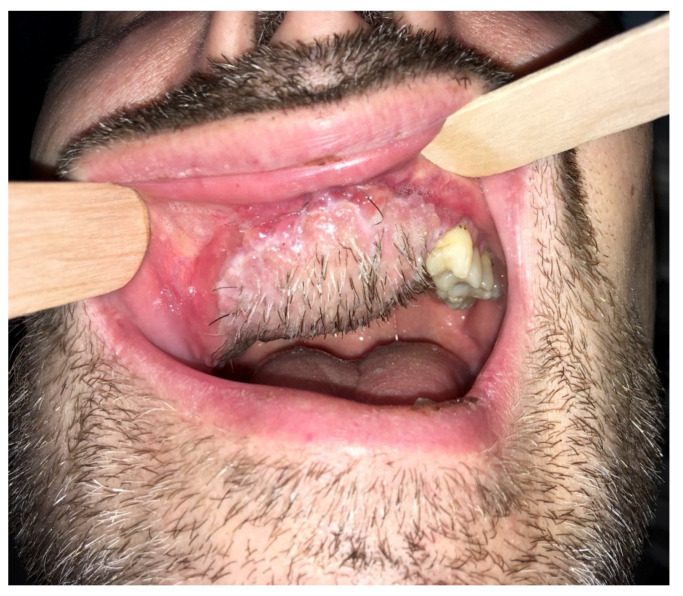
Postsurgical oral status after 45 days from surgery. The submental flap is perfectly integrated and the palate–maxillary gum completely healed in less than 60 days. Hair-bearing skin is present, which caused significant discomfort for the patient.

**Figure 7:**
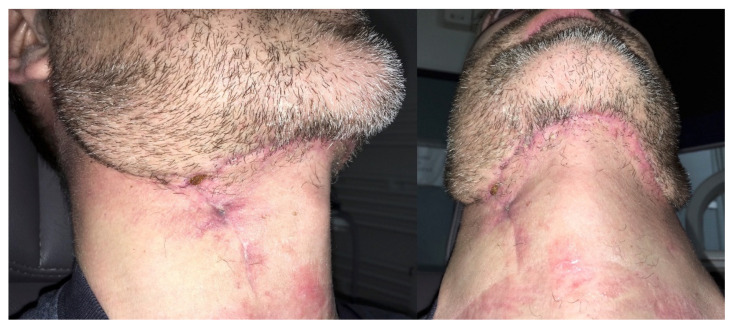
Submental and neck scar at 2 months after surgery.

**Figure 8:**
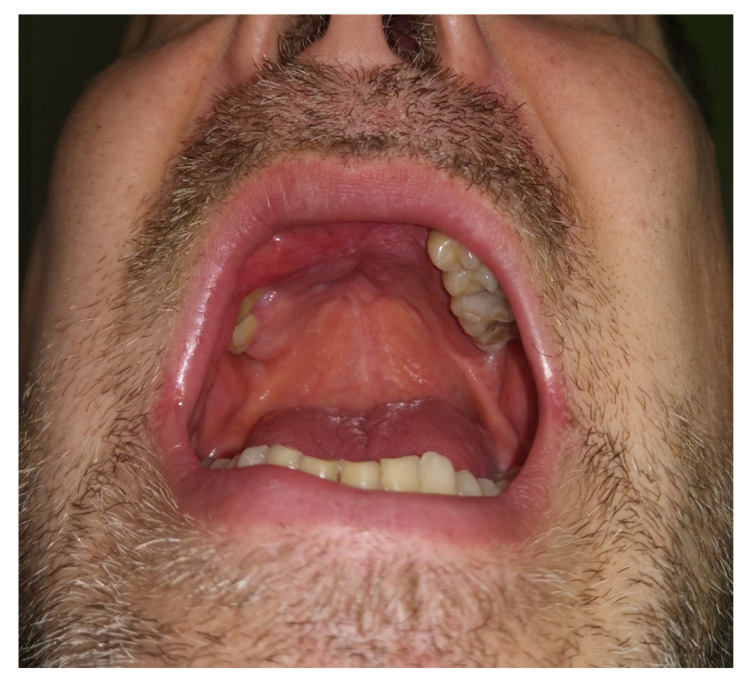
Postsurgical oral status after radiotherapy at 22 months after surgery. The flap is completely healed and no hair-bearing skin is present.

The final esthetic result was highly satisfactory in term of scars, oral reconstruction, and facial nerve function (Figure 6); healing was reached within 3 weeks, and the patient succeeded in undergoing postoperative radiotherapy within the time required (66 Gray, ended in January 2019) with no local adverse events. However, even if adjunctive therapy helped in reducing hair bearing of the reconstructive flap, tailored laser ablation sessions were scheduled, with resolution of local impairment (Figure 7 and Figure 8).

**Figure 9:**
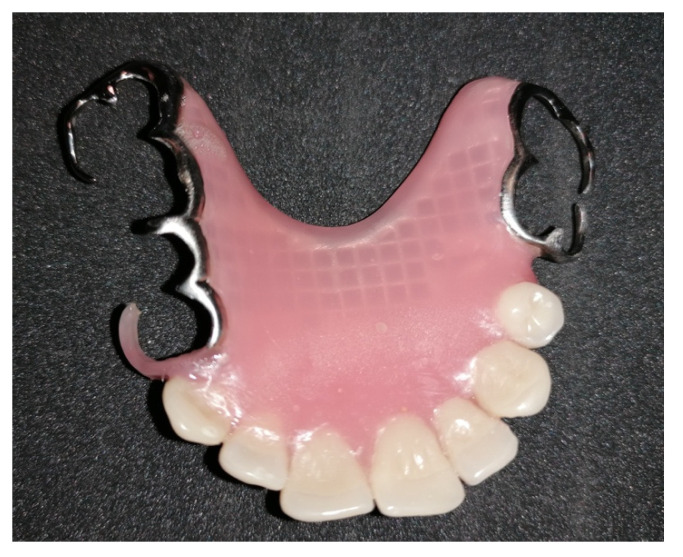
Mobile dental prosthesis with an anchoring system to the lateral teeth of the surgical defect.

At the time of writing (28 months after surgery), radiological and clinical follow-up is negative. The patient has undergone a prosthodontist evaluation, which performed a mobile prosthesis to be anchored to the lateral teeth of the surgical defect (Figure 9).

## 3. Discussion

Both cutaneous and mucosal melanomas originate from neural crest cells, which migrate as melanocyte stem cells in human skin, dermal papillae, and hair follicles [30] where they become mature melanocytes and contribute to maintaining epidermal/mucosal homeostasis [31]. Melanocytes produce melanin to protect skin from UV radiation and melatonin–serotonin, which have a role in homeostasis even if the function of mucosal melanocytes in not clear [32]. Any condition that improves proliferation of melanocytes, unlike cutaneous MM that has no apparent association with solar exposition, and chemical stimulation or trauma have been suggested as a possible cause of initial transformation towards a precancerous lesion, due to intermediate metabolites of melanogenesis, which have immunosuppressive properties and can reduce the activity of the immune system [1]. In POMMs, the chain of molecular events that induces malignant transformation is still unknown, and these lesions are associated with poor prognosis. For this reason, there is no consensus on MM treatment based on randomized trials and, as general rule, surgery is still considered the cornerstone of treatment of head and neck MM and radical tumor excision, with disease-free surgical margins, is recommended [26]. Other nonsurgical treatments such as immunotherapy can improve overall survival in a patient with cutaneous melanoma, but the efficacy of ipilimumab in patients with MM is still unknown.

In the present case, the treatment of choice was tailored surgery based on the preoperative histologic mapping. However, given the extended type of surgery performed and the need for postoperative radiotherapy, we decided to reconstruct the surgical gap in order to guarantee proper and fast healing. Such a decision is not always standardized and falls on the SIF, which is a pedicled cutaneous flap with reconstructive outcomes similar to the widely used forearm free flap [33,34,35,36]. This pedicle flap was first described by Martin et al. in 1990 [37], but it was Sterne and Hall in 1996 [38] who introduced its use in oral cavity reconstruction. The SIF is an axial patterned flap based on the submental artery, a branch of the facial artery, which arises deep to the submandibular gland. This artery runs superficial to the mylohyoid muscle and gives rise to a variable number of perforators that pierce the platysma muscle and supply dermal plexus in the area of the submental skin. The advantages of this flap include its minimal donor site morbidity, pliability, thickness, and large skin paddle in accordance with the pinch test, modest length of the pedicle up to 5 cm, with a good arch of rotation, when the entire facial artery is released. The skin is harvested in the submental area, and the width of the flap is determined by the laxity of the skin allowing primary closure, which is easier in the elderly; in men, this hair-bearing skin is very helpful in the reconstruction of the preauricular area, giving perfect camouflage, while it can be a problem in oral reconstruction. If postoperative radiotherapy is expected, beard hairs should fall down, otherwise several laser ablation sessions are needed to resolve the impairment. Moreover, extreme caution has to be applied in flap harvesting to avoid injury to the mandibular branch of the facial nerve. Relative contraindications to SIF harvesting are prior radiotherapy and the presence of metastatic lymph nodes in level IA–IB. Modifications of this flap have been described with the aim of incorporating a segment of the mandibular rim, of increasing arterial pedicle length with the reverse flow [39,40], and of increasing venous pedicle length with microvascular anastomosis (hybrid flap) [41]. As a result, this pedicled local flap is a good option for reconstructive processes of the head and neck region.

In broad terms, after ablative surgery of the hard and/or soft palate, without its reconstruction, the patient will have unintelligible hypernasal speech, difficulty chewing and swallowing, nasal regurgitation, poor masticatory function, and facial disfigurement due to loss of support for the midfacial soft tissues [42]. Surgical and nonsurgical reconstructive alternatives are possible in case of oral ablative surgery, and choices include a free flap (fibula, ALT, or scapular tip), local pedicle flap (temporal muscle), and prosthesis (palatal obturator). When the cancer does not involve more than half of the hard palate, dental prosthetic can be applied, otherwise an immediate reconstruction can be done with mucoperiosteal palatal island flap or temporalis flap. For larger surgical defects, free flap tissue transfers are needed [43]. To better define the surgical gap and the reconstruction possibilities, several classification schemes have been employed. It is noteworthy to mention Okay classification that considers four classes: class IA defect involves part of the hard palate, but not the tooth-bearing alveolus; class IB collects defects of maxillary alveolus and dentition posterior to the canines; class II defect considers involvement of any part of the alveolus and hard palate but including only one canine. When both canines are included in the surgical demolition or when a transverse palatectomy defect is larger than 50%, a Class III is considered. A reconstructive choice for each class is expected. Theoretically, our case report should be classified as a class III, because the alveolar defect included both canines, however it was a very atypical demolition of the hard palate, very limited, not full thickness, and localized to the central part. Usually nonsurgical rehabilitation of Okay class III defects is avoided because of obturator instability, caused by lack of sufficient dentition for clasping and lack of structural support from the remaining palate [44]. In these cases, an osteo-cutaneous free flap reconstruction is favored, because it allows positioning of osteo-integrated dental implants. However, in our patient, the maintenance of molar and premolar teeth and the presence of most of the palatal arch, which means structural support, permitted the application of a stable prosthesis. Other reports present in literature, regarding surgical treatment of POMM, suggest obturator prosthesis only in case of very limited demolition of the hard palate [45]. Usually, a mold of the palate is used to fabricate the obturator, which is temporarily placed in the surgical gap and fixed with stiches at the end of surgery. When surgical healing is reached, the prosthesis is held in place by natural undercuts in the defect using adhesives or clips. The advantage of this technique is that it allows direct examination of the surgical field during follow-up, but with the disadvantage of daily maintenance and dependency, which are not well tolerated in younger patients. In our case report, after radiotherapy, the patient was followed by the head and neck surgeon and prosthodontist to monitor the healing defect; only after complete palatal mucosal repair and laser ablation to stop hair-bearing skin regrowth, a mobile dental prosthesis was proposed with an anchoring system to the lateral teeth of the surgical defect.

## 4. Conclusions

MMs are uncommon lesions of the head and neck region with high mortality. Considering the lack of identified predictors of survival, best clinical practice is represented by radical tumor excision with disease-free surgical margins, eventually associated with postoperative radiotherapy. In some cases, given the large surgical gap and the need for rapid healing, surgical reconstruction is needed, and several flaps can be used. The SIF can be considered to be one of the most usable, thanks to reconstructive outcomes and local advantages.

All subjects gave their informed consent for inclusion before they participated in the study. The study was conducted in accordance with the Declaration of Helsinki, and the protocol was approved by the Ethics Committee of San Raffaele Hospital (150920).

## Data Availability

The data presented in this study are available on request from the corresponding author. The data are not publicly available due to privacy restrictions.
